# Microbially Mediated Methylation of Arsenic in the Arsenic-Rich Soils and Sediments of Jianghan Plain

**DOI:** 10.3389/fmicb.2018.01389

**Published:** 2018-07-06

**Authors:** Xian-Chun Zeng, Ye Yang, Wanxia Shi, Zhaofeng Peng, Xiaoming Chen, Xianbin Zhu, Yanxin Wang

**Affiliations:** ^1^State Key Laboratory of Biogeology and Environmental Geology, School of Environmental Studies, China University of Geosciences, Wuhan, China; ^2^School of Life Sciences, Wuchang University of Technology, Wuhan, China

**Keywords:** arsenic methylation, ArsM, dimethylarsinic acid (DMAs^V^), monomethylarsonic acid (MMAs^V^), arsenitemethylating bacteria, Jianghan Plain

## Abstract

Almost nothing is known about the activities and diversities of microbial communities involved in As methylation in arsenic-rich shallow and deep sediments; the correlations between As biomethylation and environmental parameters also remain to be elucidated. To address these issues, we collected 9 arsenic-rich soil/sediment samples from the depths of 1, 30, 65, 95, 114, 135, 175, 200, and 223 m in Jianghan Plain, China. We used microcosm assays to determine the As-methylating activities of the microbial communities in the samples. To exclude false negative results, we amended the microcosms with 0.2 mM As(III) and 20.0 mM lactate. The results indicated that the microbial communities in all of the samples significantly catalyzed arsenic methylation. The *arsM* genes were detectable from all the samples with the exception of 175 m, and 90 different *arsM* genes were identified. All of these genes code for new or new-type ArsM proteins, suggesting that new As-methylating microorganisms are widely distributed in the samples from shallow to deep sediments. To determine whether microbial biomethylation of As occurs in the sediments under natural geochemical conditions, we conducted microcosm assays without exogenous As and carbons. After 80.0 days of incubation, approximately 4.5–15.5 μg/L DMAs^V^ were detected in all of the microcosms with the exception of that from 30 m, and 2.0–9.0 μg/L MMAs^V^ were detected in the microcosms of 65, 114, 135, 175, 200, and 223 m; moreover, approximately 18.7–151.5 μg/L soluble As(V) were detected from the nine sediment samples. This suggests that approximately 5.3, 0, 8.1, 28.9, 18.0, 8.7, 13.8, 10.2, and 14.9% of total dissolved As were methylated by the microbial communities in the sediment samples from 1, 30, 65, 95, 114, 135, 175, 200, and 223 m, respectively. The concentrations of biogenic DMAs^V^ show significant positive correlations with the depths of sediments, and negative correlations with the environmental NH_4_^+^ and NaCl concentrations, but show no significant correlations with other environmental parameters, such as NO_3_^-^, SO_4_^2+^, TOC, TON, Fe, Sb, Cu, K, Ca, Mg, Mn, and Al. This work helps to better understand the biogeochemical cycles of arsenic in arsenic-rich shallow and deep sediments.

## Introduction

Arsenic is widely distributed in the Earth’s crust at an average concentration of 2.0 mg/kg. It occurs in more than 200 minerals, usually in combination with sulfur and metals (Nordstrom, 2002). Levels of arsenic differ considerably from one geographic site to another, depending on the biogeochemical conditions of the sites and the anthropogenic activities carried out in the vicinity. Arsenic deposited in sediments, rocks and minerals is usually insoluble. However, problems have arisen when the mineral arsenic was mobilized and released into groundwater (Polizzotto et al., 2008; Stuckey et al., 2016). Arsenic-contaminated groundwater exists in more than 70 countries worldwide, including Bangladesh, India, Pakistan, Burma, Nepal, Vietnam, Cambodia, China, and United States (Kirk et al., 2004; Gu and Wang, 2012; Shao et al., 2016). Natural processes, biochemical reactions, and anthropogenic activities are responsible for the dissolution and release of arsenic from minerals into groundwater (Fendorf et al., 2010).

Increasing evidences suggest that the microbial communities play major roles in the transformation, mobilization or immobilization of arsenic in the arsenic-rich sediments (Das et al., 2016; Chen et al., 2017). Diverse AOB and DARPs catalyze the redox reactions of arsenic in the sediments (Oremland and Stolz, 2003; Rhine et al., 2005; Islam et al., 2013; Kulp, 2014). AOB can oxidize As(III) into As(V) using oxygen as the terminal electron acceptor under aerobic conditions, or using nitrate, selenate, or chlorate as the terminal electron acceptor under anaerobic conditions (Silver and Phung, 2005; Stolz et al., 2006; Van der Zee and Cervantes, 2009; Kumari and Jagadevan, 2016; Zeng et al., 2016; Yang et al., 2017). As(V) can also be directly reduced into As(III) by DARPs using lactate, pyruvate, acetate, or other organic/inorganic materials as the sole electron donor (Sierra-Alvarez et al., 2005; Lear et al., 2007; Song et al., 2009; Kudo et al., 2013; Ohtsuka et al., 2013; Osborne et al., 2015). DARPs were found to be the major driver of the dissolution and release of arsenic from sediments into groundwater (Kudo et al., 2013; Ohtsuka et al., 2013; Osborne et al., 2015; Chen et al., 2017; Wang et al., 2017).

In addition to the microorganisms-catalyzed redox reactions of arsenic, it was recently found that biomethylation of arsenic occurred in the arsenic-rich sediments of the southern Willamette Basin, near the Eugene-Springfield area of Oregon, United States (Maguffin et al., 2015). An aquifer injection test indicated that tentative biomethylation could produce dimethylarsinate at a rate of approximately 0.09% of total dissolved arsenic per day, comparable to rates of dimethylarsinate production in surface environments. It was estimated that global biomethylation of arsenic in aquifers has a potential to transform 100 tons of inorganic arsenic into methylated arsenic species per year (Maguffin et al., 2015). This suggests that microbial methylation of arsenic in the arsenic-rich sediments may play a key role in the global biogeochemical cycles of arsenic. Actually, the methylarsenicals have been detected in groundwater worldwide since 1973 (Braman and Foreback, 1973; Del Razo et al., 1990; Lin et al., 1998, 2002; Shraim et al., 2002; Xie et al., 2009; Watts et al., 2010; Christodoulidou et al., 2012). However, the substantial links between arsenic methylation and microbial communities, as well as the correlations between arsenic methylation activities of indigenous microbial communities and environmental parameters in the arsenic-rich sediments remain to be elucidated.

Many bacteria, archaea, fungi, and animals are able to methylate As (Zhu et al., 2014). Arsenic methylation was catalyzed by ArsM (Ajees et al., 2012). The first *arsM* gene was cloned from the soil bacterium *Rhodopseudomonas palustris* (Qin et al., 2006). It codes for a protein with 283 amino acid residues. ArsM proteins exist in both prokaryotic and eukaryotic microorganisms (Zhu et al., 2017). Purified ArsM can convert As(III) into DMAs^V^, TMAO, and volatile TMAs^III^ (Qin et al., 2006, 2009; Slyemi and Bonnefoy, 2012; Chen et al., 2014; Zhu et al., 2014). The *arsM* gene can be used as a molecular marker for investigations of the diversities of As-methylating microbes (Jia et al., 2013). Recently, some single bacterial strains with significant As-methylating activities were isolated from paddy soils, wastewater ponds, and microbial mats (Kuramata et al., 2015; Zhang J. et al., 2015; Huang et al., 2016; Wang et al., 2016). Functional analyses suggest that ArsMs play a role in the detoxification of As(III) and could be exploited in bioremediation of arsenic-contaminated groundwater (Qin et al., 2006).

Jianghan Plain is an alluvial plain located in the middle and south of the Hubei Province, China. Geochemical surveys indicated that groundwater in some areas in Jianghan Plain was contaminated by arsenic (Gan et al., 2014). Previously, we found that DARPs widely exist in the deep sediments of this plain, and significantly catalyze the dissolution, reduction, and release of arsenic from sediments into groundwater (Chen et al., 2017). Our group also found that sulfate markedly enhances the DARPs-mediated dissolution, reduction and release of arsenic and iron from mineral phase into groundwater (Wang et al., 2017). This study aimed to explore the diversity and functions of the arsenic-methylating bacteria from the arsenic-rich sediments in Jianghan Plain.

## Materials and Methods

### Sampling

**FIGURE 1:**
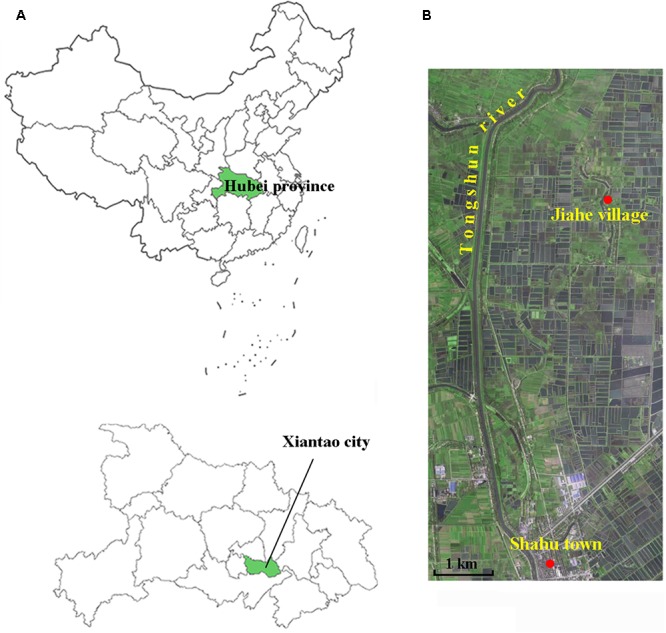
Illustrations of the sampling site. **(A)** Geographical map of the sampling location in the Jiahe village of the Shahu town in Jianghan Plain. **(B)** Satellite map of the sampling site.

The sampling site (113°36′35.028′′E, 30°8′34.944′′N) was near a paddy field, located in the Jiahe village that is affiliated to the Shahu town of Xiantao city, Hubei province, China (**Figure 1**). Xiantao area is one of the most important food production regions in China. Jiahe is a little rural village that lies in the riverside of a rivulet, approximately 1.9 km from the Tongshun River. A borehole with a depth of 230 m was drilled using the direct-mud rotary drilling technique as described elsewhere (Keely and Boateng, 1987). Soil and sediment samples were collected from the depths of 1, 30, 65, 95, 114, 135, 175, 200, and 223 m. The external layers of the sediment cores were carefully removed to avoid contaminations and oxidation by oxygen. The samples were placed in sterilized tubes that were put in an anaerobic bag buried in the ice. All of the samples were transported into the laboratory in 12.0 h.

### Geochemical Analyses

To determine the total arsenic content, one gram of dried sample powders was mixed with 20.0 mL of aqua regia. After 2.0 h of incubation at 100°C, the mixture was centrifuged at 10,000 *g*/min for 5.0 min, and the supernatant was collected for determination of the arsenic concentration using AFS (AFS-9600, Haiguang, Beijing, China). Soluble arsenic species, including total soluble As, MMAs^V^, and DMAs^V^, were determined by high-performance liquid chromatography linked to AFS (HPLC-ICP-MS) (LC-20A, Shimadzu, Japan; ELAN, DRC-e, PerkinElmer, United States). An anion-exchange column (PRP-X100, Hamilton; 4.1 mm i.d × 250 mm, 10 μm) was used for the measurement. The mobile phase consisted of 10.0 mM (NH_4_)_2_HPO_4_ and 10.0 mM NH_4_NO_3_, adjusted to pH 6.0 with 4% formic acid. The mobile phase was pumped through the column at a flow rate of 1.0 mL min^-1^.

Total organic carbon was determined with a TOC analyzer (multiN/C 3100, Analytik Jena, Germany). Concentrations of anions were measured using ion chromatography (DX-120, Dionex, United States). Concentrations of metal ions were determined using ICP-AES (IRIS Intrepid II XSP, Thermo Fisher Scientific, United States).

### Determination of As-Methylating Activities of the Sediment Samples

To detect the As-methylating activity of the microbial community from different sediment samples, active microcosms were prepared in triplicate by inoculating 8.0 g of samples into 20.0 mL of simulated groundwater (Wang et al., 2017) amended with 0.2 mM As(III) and 20.0 mM lactate in a 50-mL flask. Triplicate controls were each prepared by mixing 8.0 g of autoclaved samples with 20.0 mL of simulated groundwater. All of the mixtures were incubated at 30°C without shaking, under microaerobic conditions. After 80.0 days, approximately 1.0 mL of cultures was removed from each flask for determination of the concentrations of MMAs^V^ and DMAs^V^ using HPLC-ICP-MS.

### Methylated as Release Assay in the Absence of Exogenous as and Carbon Source

To determine if the As-methylating microbes can catalyze the release of methylated As from the arsenic-rich sediments under natural conditions, we performed microcosm assays with the sediment samples in the absence of exogenous As and organic carbon. An active microcosm was prepared by mixing 8.0 g of the sediment samples with 20.0 mL of simulated groundwater in a 50-mL flask. All of the microcosms were incubated at 30°C for 80.0 days without shaking, under microaerobic conditions. After 80.0 days of incubation, approximately 1.0 mL of slurries was removed from the flaks for measurement of the concentrations of soluble As, MMAs^V^ and DMAs^V^.

### Analysis of the Correlations Between Methylated as and Geochemical Parameters

The Spearman’s Rank Correlation Coefficient was used to determine the correlations between the concentrations of methylated As species produced by microbial communities, and the geochemical parameters in the environment. The SPSS software was used for statistical analyses. Correlations were considered to be statistically significant at a 95% confidence level (*P* < 0.05).

### Amplification, Cloning, and Analysis of *arsM* Genes From Genomic DNAs of the Samples

To detect the diversity of As-methylating microbes from the samples, three pairs of primers were used to amplify *arsM* genes from the genomic DNA of each sample. The primers were listed in **Table 1**. Metagenomic DNA was extracted from the samples using the MiniBEST Bacterial Genomic DNA Extraction Kit (TaKaRa, Japan). Nested PCR technique was used to amplify the *arsM* genes from the genomic DNA. The first run of PCR was performed using the primers arsMF1 and arsMR2; the PCR products from the first run were purified and used as templates for the second run with the primers arsMF2 and arsMR1 or arsMF3 and arsMR1. The predicted lengths of the PCR products using the three sets of primers are 340, 230, and 210 bp, respectively. PCR amplifications were carried out in a reaction volume of 50 μL containing 2 μL of bacterial genomic DNA as template, 1.0 unit of Taq DNA polymerase (TaKaRa, Japan), 10 pmol of each primers and 0.2 mM dNTPs. Reaction conditions were as follows: pre-denaturing at 94°C for 5 min, 30 cycles of 94°C for 30 s, 65°C for 30 s, and 72°C for 30 s, and ended with a final extension phase at 72°C for 10 min. PCR products were gel purified using the E.Z.N.A gel extraction kit (Omega, United States). Purified DNA was ligated into a T vector. An *arsM* gene library was generated by introducing the recombinant plasmids into *Escherichia coli* DH5α competent cells. All of the clones from the library were sequenced and analyzed as described previously (Zhong et al., 2017).

**Table 1:** PCR primers employed in this study.

Target	Primer name	Length	Position^a^	Reference
*arsM*	arsMF1: TCYCTCGGCTGCGGCAAYCCVAC	23	187–209	Jia et al., 2013
	arsMF2: GTGCTCGAYCTSGGCWCCGGC	21	241–261	Jia et al., 2013
	arsMF3: GGCATCGACGTGCTKCTBTCSGC	23	319–341	Jia et al., 2013
	arsMR1: AGGTTGATGACRCAGTTWGAGAT	23	451–473	Jia et al., 2013
	arsMR2: CGWCCGCCWGGCTTWAGYACCCG	23	511–533	Jia et al., 2013
	arsMR3: GCGCCGGCRAWGCAGCCWACCCA	23	611–633	Jia et al., 2013
T vector	M13-47: CGCCAGGGTTTTCCCAGTCACGAC	24	–	Wang et al., 2017
	RV-M: GAGCGGATAACAATTTCACACAGG	24	–	Wang et al., 2017

^a^*The position in the arsM gene of Rhodopseudomonas palustris CGA009*.

The obtained different *arsM* genes were translated into amino acid sequences using the ExPASy server. A protein sequence homology search against the GenBank database was performed using the BLAST server. Multiple sequence alignments were conducted using the ClustalW2 software as described elsewhere (Zhang et al., 2015; Mu et al., 2016a). A phylogenetic tree of the obtained ArsMs and their closely related homologues was constructed using the maximum likelihood method implemented in MEGA 6.0 as described previously (Xu et al., 2014; Mu et al., 2016b).

### Accession Number(s)

The *arsM* sequences from the Jianghan Plain have been deposited into the GenBank database under the accession numbers MH177487 to MH177576.

## Results

### Characterization of the Sampling Site

We collected nine sediment samples from the depths of 1, 30, 65, 95, 114, 135, 175, 200, and 223 m, respectively. Geochemical analyses indicated that the sediments contain high contents of total As (ranging from 6.74 to 42.11 mg/kg) and soluble As (ranging from 1.9 to 100.7 μg/L). The arsenic contents show no correlations with the depths of the sediments. The concentrations of total arsenic also show no correlations with those of soluble arsenic; this suggests that multiple factors controlled the arsenic dissolution and release from the sediments. The sediment samples contain 0.27–8.37 g/kg TOC and 0.18–1.26 g/kg TON; these substances could provide essential carbon and nitrogen sources for the growth of microorganisms in the sediments. The sediments also contain relatively high contents of sulfate (ranging from 14.53 to 863.87 mg/kg), and relatively low contents of ammonium (ranging from 3.12 to 52.77 mg/kg) and nitrate (ranging from 0.23 to 3.03 mg/kg).

### As-Methylating Activities of the Microbial Communities From the Sediments

**FIGURE 2:**
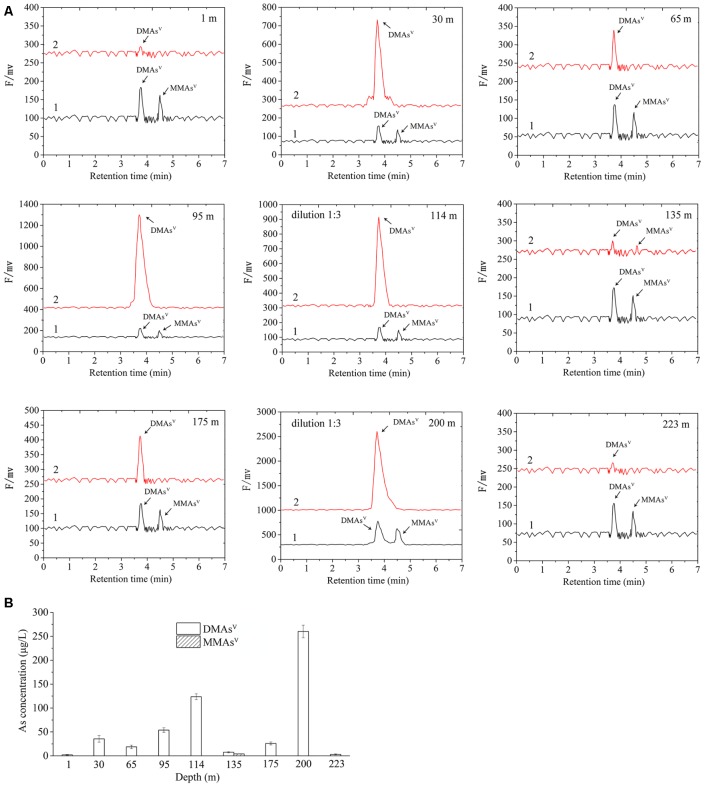
Arsenic methylation activities of the microbial communities of the nine samples collected from the arsenic-rich shallow and deep sediments in Jianghan Plain. **(A)** HPLC-ICP-MS chromatograms of the methylated As species produced by the microbial communities of the samples from 1 to 223 m after 80.0 days of incubation. Curve 1, standards of DMAs^V^ and MMAs^V^; curve 2, DMAs^V^ and MMAs^V^ concentrations in the microcosms amended with 0.23 mM As(III) as the substrates of ArsM enzymes, and 20.0 mM lactate as the carbon source after 80.0 days of incubation. **(B)** Concentrations of the methylated As in the microcosms of the samples from 1 to 223 m after 80.0 days of incubation.

Microcosm assay was used to determine the As-methylating activities of the nine sediment samples. The microcosms were amended with 0.23 mM As(III) as the substrate of ArsM enzymes, and 20.0 mM lactate as the carbon source. The results showed that no detectable amounts of methylated arsenic species were observed in the autoclaved sediment slurries. In contrast, when the sediment microcosms were not autoclaved, 2.06, 35.53, 18.83, 53.99, 121.86, 7.54, 7.00, 260.38, and 3.02 μg/L DMAs^V^ were detected in the microcosms from 1, 30, 65, 95, 114, 135, 175, 200, and 223 m, respectively (**Figures 2A,B**). No significant amounts of MMAs^V^ were detectable in all of the sediment samples with the exception of that from the depth of 135 m, in which 3.30 μg/L of biogenic MMAs^V^ was produced. This suggests that the microbial communities in the nine sediment samples were able to significantly catalyze As methylation, and the dominant products were DMAs^V^.

### Methylated as Release From the Sediments

**FIGURE 3:**
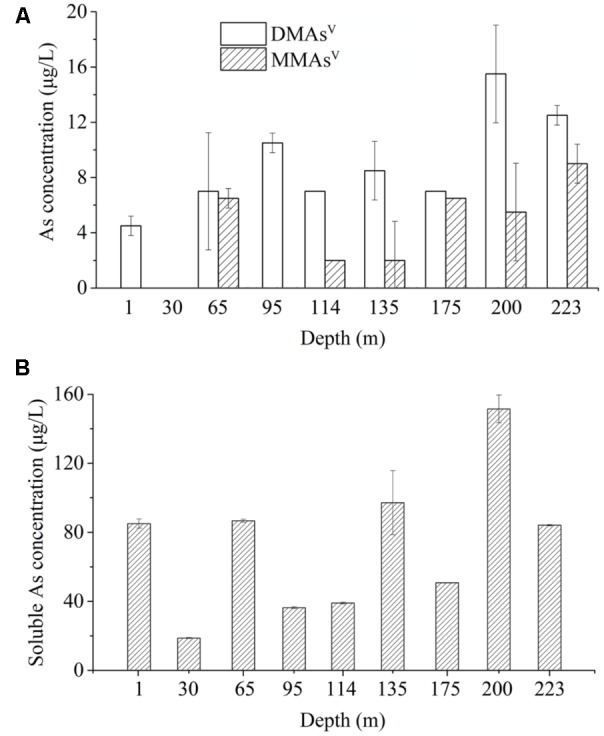
The microbial communities-catalyzed methylation and dissolution of arsenic in the microcosms prepared from the sediment samples of the depths 1 to 223 m in the absence of exogenous As and carbon source. **(A)** Concentrations of biogenic methylated As after 80.0 days of incubation. **(B)** Concentrations of biogenic soluble As after 80.0 days of incubation.

To determine if the As-methylating microbes catalyze the release of methylated As from the arsenic-rich sediments under natural conditions, we performed methylated As release assay using the microcosms in the absence of any exogenous organic carbon and As(III) compounds. We found that 4.5, 7.0, 10.5, 7.0, 8.5, 7.0, 15.5, 12.5 μg/L DMAs^V^ were released from the slurries of the samples from the depths 1, 65, 95, 114, 135, 175, 200, and 223 m, respectively, and no significant amount of DMAs^V^ were detected from the microcosm of 30 m (**Figure 3A**). In contrast, 6.5, 2.0, 2.0, 6.5, 5.5, 9.0 μg/L MMAs^V^ were detected in the slurries of the samples from the depths 65, 114, 135, 175, 200, and 223 m, respectively, and no detectable MMAs^V^ was found from the slurries of other sediment samples. This suggests that significant amount of DMAs^V^ were generated in the sediment samples after 80.0 days of incubation without shaking, even though no exogenous carbons and As(III) were added to the microcosms.

We also examined the concentrations of soluble As in the sediment samples after 80.0 days of incubation. The results showed that approximately 85.05, 18.69, 86.64, 36.36, 39.00, 97.20, 50.79, 151.53, and 84.09 μg/L of soluble As(V) were detected in the slurries of the samples from 1, 30, 65, 95, 114, 135, 175, 200, and 223 m, respectively (**Figure 3B**). This suggests that 5.29, 0, 8.08, 28.87, 17.95, 8.74, 13.78, 10.23, and 14.87% of total soluble As were methylated by the microbial communities in the microcosms of the samples from 1, 30, 65, 95, 114, 135, 175, 200, and 223 m, respectively.

### Correlations of the Methylated as Species and Some Geochemical Parameters

**FIGURE 4:**
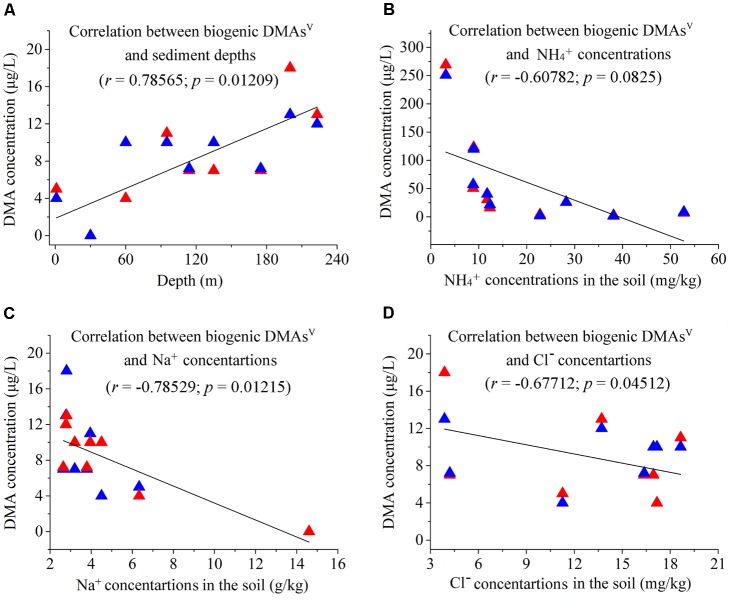
Correlations between arsenic biomethylation and some environmental parameters. The red and blue triangles represent two different assays. **(A)** Correlation between biogenic DMAs^V^ and sediment depths (*r* = 0.78565, *p* = 0.01209); **(B)** Correlation between biogenic DMAs^V^ and concentrations of environmental NH_4_^+^ (*r* = –0.60782, *p* = 0.0825); **(C)** Correlation between biogenic DMAs^V^ and concentrations of environmental Na^+^ (*r* = –0.78529, *p* = 0.01215); **(D)** Correlation between biogenic DMAs^V^ and concentrations of environmental Cl^-^ (*r* = –0.67712, *p* = 0.04512).

The Spearman’s Rank Correlation Coefficient was used to determine the correlations between methylated As species and geochemical parameters. We found that the concentrations of biogenic DMAs^V^ from the sediments of different depths show significant positive correlations with the depths of the sediments (*r* = 0.78565; *p* = 0.01209) (**Figure 4A**), and negative correlations with the contents of NH_4_^+^ (*r* = -0.60782; *p* = 0.0825), Na^+^ (*r* = -0.78529; *p* = 0.01215), and Cl^-^ (*r* = -0.67712; *p* = 0.04512) in the environment (**Figures 4B–D**).

In comparison, the concentrations of biogenic DMAs^V^/MMAs^V^ show no significant correlations with other geochemical parameters, including TOC (*r* = 0.19646; *p* = 0.61243), TON (*r* = -0.0307; *p* = 0.9375), Sb (*r* = -0.30677; *p* = 0.422), Fe (*r* = -0.30099; *p* = 0.43126), Cu (*r* = -0.2516; *p* = 0.51372), K (*r* = -0.27796; *p* = 0.46895), Ca (*r* = 0.41564; *p* = 0.26588), Mg (*r* = 0.08841; *p* = 0.82105), Mn (*r* = 0.47185; *p* = 0.19972), Al (*r* = -0.21077; *p* = 0.58619), NO_3_^-^ (*r* = 0.28976; *p* = 0.44946), and SO_4_^2-^ (*r* = -0.08394; *p* = 0.83).

### Diversities of As-Methylating Microbes in the Sediment Samples

To understand the microbial basis of the As-methylating activities of the nine sediment samples, we explored the diversities of the As-methylating microbes present in the microbial communities by cloning, sequencing and analyzing the *arsM* genes from the metagenomic DNAs of the samples. The *arsM* genes were detectable from all of the samples with the exception of the sample from the depth of 175 m. A total of 524 clones were obtained from the DNA libraries, including 83, 100, 53, 49, 47, 95, 49, and 48 clones from the samples of the depths 1, 30, 65, 95, 114, 135, 200, and 223 m, respectively. All of the clones were picked for sequencing. We identified 90 different ArsM proteins from the microbial communities of the eight samples, including 20, 23, 8, 10, 4, 5, 13, and 7 different ArsMs from the samples of 1, 30, 65, 95, 114, 135, 200, and 223 m, respectively (Supplementary Figure S1). The lengths of these *arsM* genes are either 233 bp (for the genes from the depths 1, 65, 95, 135, 200, and 223 m) or 209 bp (for the genes from the depths of 30 and 114 m). They share 25–98% sequence identities with each other. This suggests that the As-methylating microbes widely exist in the arsenic-rich shallow and deep sediments. We did not detect any *arsM* gene from the genomic DNA of the sample from 175 m; this does not mean that there are no *arsM* genes in the microbial community of the sample. It is most likely that the primers used in this study is not complementary to the specific regions of the *arsM* genes from the sample of 175 m.

**FIGURE 5:**
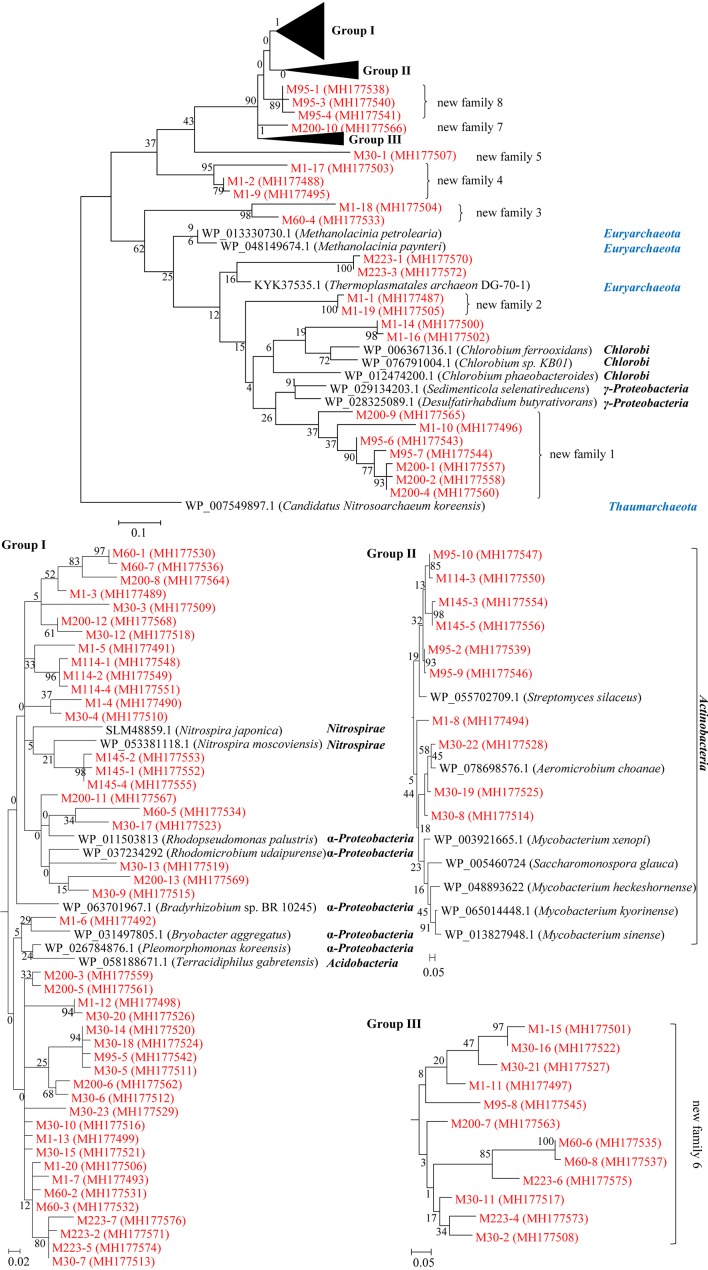
Characterization of the ArsM proteins identified from microbial communities of the sediment samples from Jianghan Plain. Maximum likelihood tree constructed from the multiple sequence alignments of the obtained ArsM proteins from the sediment samples and their closely related homologues from other known microorganisms. All of the ArsM sequences in this study are shown in red; numbers on the branches are bootstrap values based on 1000 replicates; the scale bar represents the average number of substitutions per site.

The amino acid sequences of the obtained ArsM proteins were each used as queries to search against the GenBank database using the BLAST sever. We found that these ArsMs share 35–98% sequence homology with other known ArsMs from bacteria and archaea. A phylogenetic tree was constructed based on the multiple alignments of the ArsM proteins from this study and their closely related ArsMs from other known microorganisms (**Figure 5**). An ArsM sequence from archaea was chosen as the outgroup.

If a group of ArsMs from the sediments shares less than 60% maximal homology with other known ArsMs from microorganisms, and they form an independent cluster in the tree, we classified them as a new subfamily of ArsMs. The tree illustrates that we identified eight novel subfamilies of ArsM proteins from the sediments of Jianghan Plain: subfamily 1 (M200-4, M200-2, M200-1, M95-7, M95-6, M1-10, M200-9), 2 (M1-19, M1-1), 3 (M60-4, M1-18), 4 (M1-9, M1-2, M1-17), 5 (M30-1), 6 (M30-2, M223-4, M30-11, M223-6, M60-8, M60-6, M200-7, M95-8, M1-11, M30-21, M30-16, M1-15), 7 (M200-10), and 8 (M95-4, M95-3, M95-1) (**Figure 5**). Other ArsM proteins from this study are clustered together with ArsMs from the bacterial taxa γ*-Proteobacteria*, *Chlorobi*, *Acidobacteria*, α*-Proteobacteria*, *Nitrospirae*, and archaea *Euryarchaeota*. This highlights the diversities of the As-methylating microorganisms from the arsenic-rich sediments in Jianghan Plain.

It is interesting to see that ArsM proteins M200-4, M200-2, M200-1, M95-7, M95-6, M1-10, M200-9, M1-16, M1-14, M1-19, M1-1, M223-3, M223-1 are affiliated to the cluster that is located between the ArsMs from archaea *Thaumarchaeota* and *Euryarcheota*; this suggests that these arsenic-methylating microbes belong to archaea, or bacteria acquired *arsM* genes from archaea by gene horizontal transfer in the sediments.

## Discussions

Global arsenic transformation includes the redox reactions, and methylation/thiolation cycles (Zhu et al., 2014). Despite the fact that As is toxic to microorganisms, diverse prokaryotes and eukaryotes obtain their energy from oxidation of As(III), or reduction of As(V). Some microorganisms also detoxify As by oxidizing As(III) to less toxic As(V), or by using *arsC* gene operon capable of converting intracellular As(V) into As(III) that is further transported out of the cell by an energy-dependent efflux pathway (Slyemi and Bonnefoy, 2012).

As methylation is regarded as another As detoxification mechanism for both prokaryotic and eukaryotic microbes. However, some compounds of the pathway were shown to be more toxic than the inorganic forms of arsenic (Li H. et al., 2016). It is likely that the production of MMAs(III) or DMAs(III) doesn’t only have a role in detoxification, but also acts as a primordial antibiotic to attack other species (Li J. et al., 2016). Biomethylation of As is widespread in nature, and has been widely observed in bacteria, archaea, fungi, algae, plants, animals, and humans (Zhu et al., 2014). It was estimated that biological methylation of arsenic causes approximately 2.1 × 10^7^ kg As per year to be volatilized and released into the atmosphere (Srivastava et al., 2011; Mestrot et al., 2013).

During the last decade, the investigations of microbial As methylation have focused on three aspects: (i) environmental diversity of *arsM* genes; (ii) isolation and characterization of cultivable As-methylating bacteria; (iii) construction of engineered bacterial strains expressing high levels of *arsM* genes, for bioremediation of As contaminations in the environment. As-methylating bacteria widely exist in As-contaminated paddy soils and rice rhizosphere (Jia et al., 2013; Zhao et al., 2013; Zhang S.Y. et al., 2015; Reid et al., 2017), activated sludges (Cai et al., 2013), contaminated aquatic ecosystems (Desoeuvre et al., 2016), copper mine (Xiao et al., 2016), composting manure (Zhai et al., 2017), and estuarine wetland (Zhang et al., 2017). It was found that there is a significant correlation between *arsC* and *arsM* genes in the paddy soils; this suggests that the two genes coexist well in the microbial As resistance system (Zhang S.Y. et al., 2015). Until now, at least seven cultivable As-methylating bacteria, including *Clostridium* sp. BXM, *Rhodopseudomonas palustris*, *Arsenicibacter rosenii*, *Cytophagaceae*. sp. SM-1, *Shewanella oneidensis* MR-1, *Pseudomonas alcaligenes* NBRC14159, and *Streptomyces* sp.GSRB54, have been isolated from different As-contaminated environments (Qin et al., 2006; Kuramata et al., 2015; Wang et al., 2015, 2016; Zhang J. et al., 2015; Huang et al., 2016, 2017). They possess significant As-methylating activities under aerobic, anaerobic, or microaerobic conditions. Moreover, several engineered As-methylating bacterial strains expressing high levels of ArsM proteins, such as *Pseudomonas putida* and *Bacillus subtilis*, were constructed for bioremediation of As-containing soils and organic manure (Chen et al., 2013; Huang et al., 2015); however, all these efforts were in the laboratory.

Recently, it was found that microbial methylation of arsenic could also occur in the arsenic-rich aquifers of the depths 20–40 m in the Southern Willamette Basin, Oregon, United States (Maguffin et al., 2015). However, little is known about the distributions, diversities, and activities of As-methylating microbes in the arsenic-rich sediments from different depths. In this study, we found that there is a large diversity of As-methylating microorganisms distributed in the arsenic-rich sediments from the depths of 1–223 m in Jianghan Plain in the central China. All of the sediment samples possess significant As-methylating activities. We also found that the microbial communities in the sediments catalyzed As dissolution and methylation in the absence of exogenous carbon source and As. To the best of our knowledge, this is the first report on the diversities and functions of As-methylating microbes from arsenic-rich sediments. This work also provided direct evidences that the microbial communities in the deep sediments extensively involve in global arsenic methylation reactions.

As shown in Supplementary Figure S1, we failed to detect the presence of *arsM* gene in the sample from the depth of 175 m; however, the As-methylating activity was really detectable in this sample (**Figure 3A**). We also observed that the diversities of the *arsM* genes are not always consistent with the concentrations of the methylated As in different samples. This inconsistence should be attributed to that there are other *arsM*-like genes that we failed to detect because of mismatch by the primers used for PCR amplifications.

It is interesting to see that the concentrations of biogenic DMAs^V^ from the sediments show significant positive correlations with the depths of the sediments. This suggests that pressure may be beneficial to the microbial As-methylating reactions. We also found that the concentrations of DMAs^V^ generated by microbial reactions show significant negative correlations with those of NaCl and NH_4_^+^ in the sediments. However, the mechanism for this observation remains to be elucidated. Because the civil wastewater always contains high concentrations of NaCl and NH_4_^+^, it can be inferred that anthropogenic activities could decrease the As methylation in the sediments. Considering that the sampling site of this study was located in a paddy field, and the groundwater in the area contains high concentrations of NH_4_^+^ (Gan et al., 2014), it is no surprise that the groundwater in Jianghan Plain contained little methylated As.

As shown in **Table 2** and **Figure 3B**, a comparison between the soluble As in the original sediment samples and in the incubated samples indicated that in some cases, the latter is higher, in other cases, it is lower. This observation was attributed to that the microbial communities from different depths have different As mobilization/immobilization activities.

**Table 2:** Geochemical features of the nine samples from the Jianghan Plain.

Parameters	Sediment samples															
	1 m	30 m	65 m	95 m	114 m	135 m	175 m	200 m	223 m
pH	7.24	7.25	7.11	7.19	7.29	7.41	7.37	7.35	7.67
EC (μS × cm^-1^)	242	267	250	289	367	241	213	223	271
TOC (g/kg)	1.4	0.56	0.51	0.32	8.37	0.69	0.30	0.27	1.62
TON (g/kg)	1.26	0.40	0.65	0.23	1.05	0.18	0.22	0.30	0.21
Total As (mg/kg)	7.60	8.00	30.42	23.41	42.11	9.70	9.90	6.74	10.48
Soluble As (μg/L)	13.9	100.7	9.5	1.9	41.9	2.0	17.7	30.5	14.5
SO_4_^2-^ (mg/kg)	14.53	40.62	863.87	422.46	532.11	446.11	115.67	98.31	94.58
NO_3_^-^ (mg/kg)	0.59	1.91	0.42	0.39	0.58	3.03	2.18	2.55	0.23
NH_4_^+^ (mg/kg)	38.10	11.76	12.32	8.84	8.94	52.77	28.22	3.12	22.76
Cl^-^ (mg/kg)	11.26	511.76	17.17	18.65	16.37	16.95	4.21	3.88	13.71
K (g/kg)	26.40	17.65	11.42	9.32	13.98	9.03	9.27	11.44	20.44
Na (g/kg)	6.33	14.60	4.50	3.95	3.79	3.20	2.64	2.80	2.77
Ca (g/kg)	6.80	12.56	32.34	19.95	23.46	24.75	18.04	26.55	10.30
Mg (g/kg)	12.22	7.40	3.91	6.65	6.00	7.51	6.30	7.75	2.33
Fe (g/kg)	62.50	23.23	18.64	48.29	20.48	34.68	17.44	16.68	12.13
Mn (g/kg)	1.07	0.34	0.42	1.59	0.46	1.03	2.23	2.28	0.28
Al (g/kg)	99.15	53.10	22.89	26.75	27.11	29.78	27.32	33.52	28.33
Cu (mg/kg)	40.70	8.90	4.80	12.80	6.80	9.70	8.30	9.40	11.00
Sb (mg/kg)	1.20	0.40	0.50	0.20	0.40	0.20	0.20	0.20	0.30

Arsenic-rich groundwater is widely distributed in more than 70 countries in the world. Our work clearly indicated that As-methylating microorganisms ubiquitously exist in the arsenic-rich sediments from 1 to 223 m, and possess apparent biomethylation activities. This suggests that the microbial communities in the arsenic-rich sediments play important roles in the global transformations of inorganic As into organic forms, and the amount of released methylated As from the sediments as predicated by Maguffin et al. (2015), may be significantly underestimated; however, many *in situ* experiments are required to achieve accurate prediction.

**FIGURE 6:**
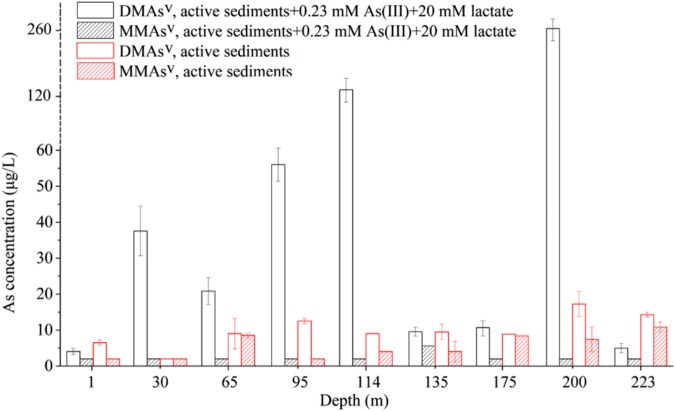
A comparison between the As-methylating activities in the presence and absence of exogenous As and organic carbon.

As shown in **Figure 6**, a comparison between the As-methylating activities in the presence or absence of exogenous As and organic carbon indicated that addition of exogenous As and C markedly stimulated the methylation activities of the microbial communities in the sediments from the depths of 30, 65, 95, 114, 175, and 200 m. In comparison, exogenous As and C inhibited the microbial arsenic-methylating activities of the sediments from other depths; this could be attributed to that the supplemented As and C dominantly promoted the growth of non-As-methylating microbes in the sediments, which competitively inhibited the growth of As-methylating microbes.

**FIGURE 7:**
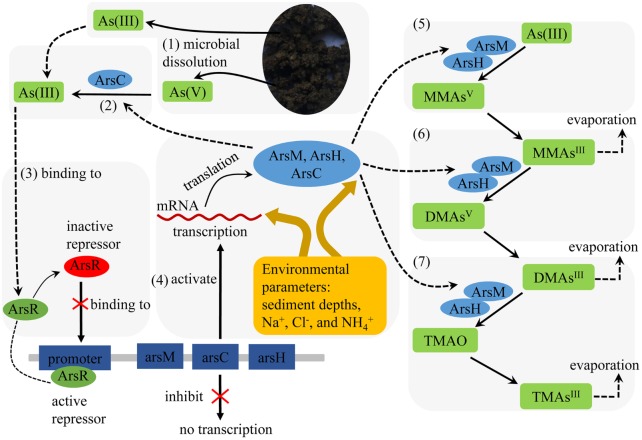
Schematic representation of the metabolic pathway for arsenic biomethylation reactions in the arsenic-rich sediments.

Based on the data of this study and related knowledge (Carlin et al., 1995; Qin et al., 2006; Ye et al., 2012; Yang and Rosen, 2016), we proposed a conceptual model for the microbes-catalyzed methylation of As in the arsenic-rich sediments (**Figure 7**). The microbial reactions started from microbial communities-catalyzed weathering, dissolution and release of As(III) and As(V) from the sediments under microaerobic conditions (1). Bacterial ArsC catalyzes the reduction of As(V) into As(III) (2).The released and produced As(III) binds to the repressor (ArsR) of bacterial *arsM-arsC-arsH* gene operon (3), and thus activate the expression of the *arsM, arsC* and *arsH* genes (4). ArsM catalyzes As methylation by converting As(III) into MMA^V^ (5) that is further converted into DMAs^V^ via reduction and methylation reactions catalyzed by ArsH and ArsM (6). Finally, DMAs^V^ is converted into TMA and TMAO by ArsM and ArsH (7).

## Conclusion

Methylation of As plays important roles in the global biogeochemical cycles of arsenic. However, little is known about whether biomethylation of As occurs in arsenic-rich shallow and deep sediments; the activities and diversities of microorganisms involved in As methylation in the sediments also remain to be elucidated. In this study, we found that the microbial communities from the sediments of 1–223 m in Jianghan Plain have efficient As-methylating activities. They can significantly catalyze the dissolution and methylation of As in the absence of exogenous As and carbon source. Functional gene analyses indicated that there are large diversities of novel As-methylating microbes widely existing in all of the samples from 1 to 223 m sediments with the exception of 175 m. The concentrations of biogenic DMAs^V^ show significant positive correlations with the depths of sediments, and negative correlations with the environmental NH_4_^+^ and NaCl. This suggests that anaerobic or microaerobic conditions and pressure could be favorable for the microbial biomethylation reactions of As. These findings for the first time provided direct evidences that the microbial communities in the arsenic-rich shallow and deep sediments catalyze arsenic methylation, and thus play key roles in the global transformation of arsenic from inorganic to organic form. The data of this study also strongly suggest that the global arsenic methylation in the arsenic-rich sediments may be significantly underestimated because previous investigation only focused the biomethylation occurred in the sediments from the depths 20–40 m. This work also is useful for us to better understand the biogeochemical cycles of arsenic in the arsenic-rich sediments.

## Author Contributions

YY, WS, ZP, XC, and XZ conducted the experiments. X-CZ, YY, WS, and YW analyzed the data. X-CZ conceived and designed the experiments, wrote the paper, contributed the reagents, materials, and analysis tools.

## Conflict of Interest Statement

The authors declare that the research was conducted in the absence of any commercial or financial relationships that could be construed as a potential conflict of interest.

## Abbreviations

AFS: atomic fluorescence spectrometry
AOB: arsenite-oxidizing bacteria
ArsM: As(III)-S-adenosylmethionine methyltransferase
DARPs: dissimilatory arsenate-respiring prokaryotes
DMAs^V^: dimethylarsinic acid
EC: electrical conductivity
HPLC-ICP-MS: high-performance liquid chromatography linked to inductively coupled plasma-mass spectrometry
ICP-AES: inductively coupled plasma-atomic emission spectrometry
MMAs^V^: monomethylarsonic acid
TMAO: trimethylarsine oxide
TMAs^III^: trimethylarsine
TOC: total organic carbon
TON: total organic nitrogen.
