# Supplementary material for: Microbially Mediated Methylation of Arsenic in the Arsenic-Rich Soils and Sediments of Jianghan Plain
Source: Front Microbiol. 2018 Jul 6;9:1389. doi: 10.3389/fmicb.2018.01389 (PMC6043643; doi:10.3389/fmicb.2018.01389)
Supplement: Supplementary file 1 [file Image_1.pdf]

Supplementary Material

Microbially Mediated Methylation of Arsenic in the Arsenic-rich Soils and Sediments of Jianghan Plain

Xian-Chun Zeng *, Ye Yang, Wanxia Shi, Zhaofeng Peng, Xiaoming Chen, Xianbin Zhu, Yanxin Wang

* **Correspondence:** Xian-Chun Zeng: xianchun.zeng@gmail.com; xianchun_zeng@hotmail.com.

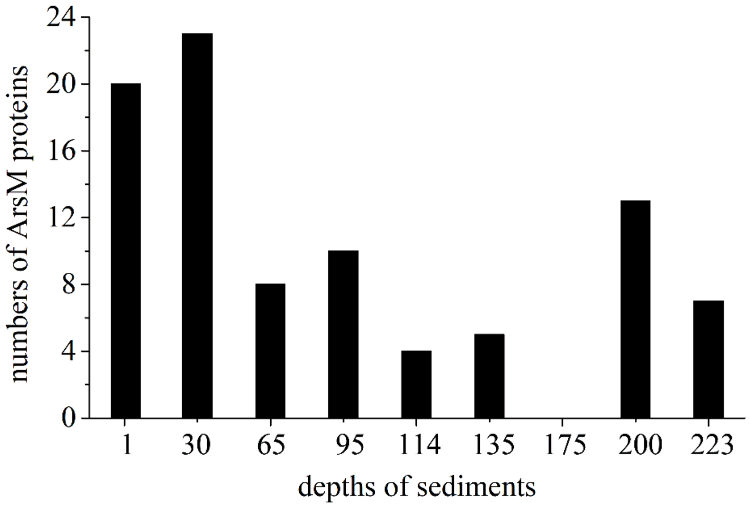

Figure S1. The numbers of different ArsM proteins identified from the microbial community of each sample from the depths of 1-223 m.
